# The Youngest Case of Metachronous Bilateral Acinic Cell Carcinoma of the Parotid Gland: A Case Report and Literature Review

**DOI:** 10.1155/2022/8474741

**Published:** 2022-05-24

**Authors:** Raid Alhayaza, M. Anas Dababo, Suresh Velagapudi

**Affiliations:** ^1^College of Medicine, Alfaisal University, Riyadh, Saudi Arabia; ^2^Department of Pathology and Laboratory Medicine, King Faisal Specialist Hospital and Research Centre, Riyadh, Saudi Arabia; ^3^Department of Otolaryngology, King Faisal Specialist Hospital and Research Centre, Riyadh, Saudi Arabia

## Abstract

**Introduction:**

Acinic cell carcinoma (ACC) is a low-grade malignant salivary neoplasm that represents 17% of all salivary gland malignancies. It has a tendency to affect young individuals, especially females. ACC mainly originates in the parotid gland and has a potential for recurrence and metastases. Rarely, ACC can affect both parotid glands in a single individual. A bilateral ACC of the parotid gland could either present as a synchronous or a metachronous tumor. *Case Report.* Our patient is a 19-year-old female known case of ACC of the right parotid gland. The tumor was resected in December 2017. After 3 years, she presented with a left parotid pain and swelling, which raised the suspicion of a contralateral metachronous tumor of the left parotid gland. In September 30, 2020 we proceeded with ultrasound-guided fine needle aspiration of the left intraparotid lesion, and the results turned out to be consistent with ACC. Here, we report a case of a 19-year-old female presenting with metachronous bilateral ACC of the parotid gland with an interval of 3 years, which is the 6^th^ of its kind in the literature and the youngest amongst them.

**Conclusion:**

Despite the rareness of metachronous occurrence of bilateral ACC of the parotid gland, it is still encountered in the medical practice. Here, we are highlighting the importance of follow-up with a periodic clinical and radiological examinations, bearing in mind the contralateral nonaffected parotid gland.

## 1. Introduction

Salivary gland tumors are considered to have various biological behaviors and clinicopathologic features [1, 2]. 17% of primary malignant salivary gland tumors are acinic cell carcinoma (ACC), a low-grade malignant salivary neoplasm. ACC predominantly occurs in young individuals, where patients under the age of 30 represent more than 16% of all cases, majority of whom are females. The parotid gland is the main site of ACC origin, in the head and neck region. It is characterized by cytoplasmic zymogen secretory granules, which is illustrated in the acinar cell serous differentiation. ACC is one of the tumors that can be associated with lymphoid-rich stroma as in this case. It has some identified risk factors such as familial predisposition and a history of radiation exposure. Moreover, ACC mostly present as a slowly enlarging lesion in the parotid gland tail. Usually, fine needle aspiration (FNA) biopsy is used to confirm the diagnosis. On the other hand, surgical excision is performed as the main treatment strategy [1]. Furthermore, radical parotidectomy is generally used in the treatment of malignant parotid tumors. Also, superficial parotid lobectomy can be used when ACC is confined to the superficial lobes and no cervical metastases were detected [3, 4]. Other modalities of treatment such as radiotherapy may be used as an adjuvant treatment based on close resection margins and perineural and perivascular invasion in certain cases. ACC has a potential for recurrence and metastases to the lungs and cervical lymph nodes and may evolve aggressively, signifying the importance of regular follow-up for these patients posttreatment [1]. Here, we report the youngest case to develop metachronous bilateral ACC of the parotid gland, which is a rare occurrence.

## 2. Case Presentation

**Figure 1:**
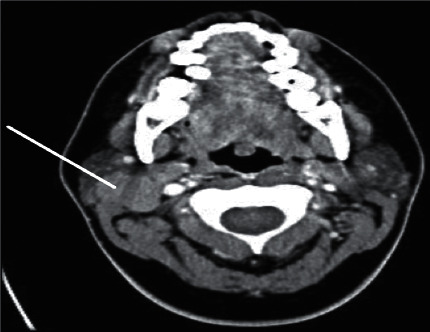
The CT scan showing a right parotid gland lesion in 2017.

**Figure 2:**
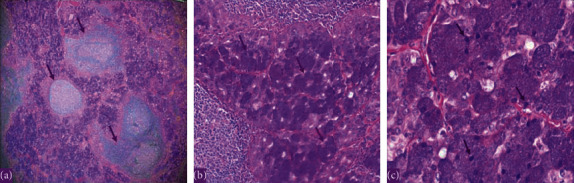
The histopathology with hematoxylin and eosin (H&E) stain showing a multifocal acinic cell carcinoma of the right parotid gland in 2017. (a) Low power showing lymphoid-rich stroma with lymphoid follicles and prominent germinal centers within tumor. (b) Medium power showing tumor with acini and granular cytoplasm. (c) High power x60 showing the basophilic granular cytoplasm (zymogen granules of neoplastic serous acini).

**Figure 3:**
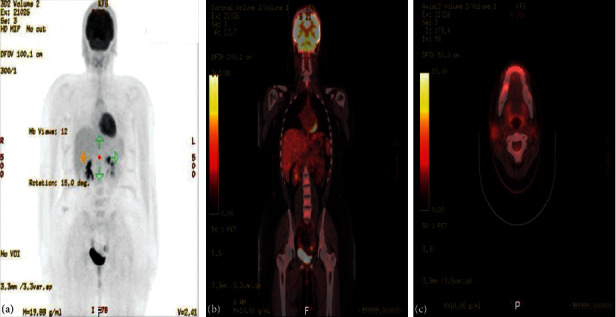
The PET scan in November 2017. (a)–(c) There is mild background level FDG avidity (SUVmax 2.2) in the region of the right parotid. This uptake is most likely postsurgical. Also, there is mild FDG avidity (SUVmax 1.7) within a few prominent (up to 11 mm short axis) right upper cervical (levels II-III) lymph nodes. The exact etiology remains indeterminate (i.e., whether could be malignant or reactive due to her recent surgery). Also, small volume lymph nodes (measuring up to 8 mm short axis) are present in the left upper cervical/level II region, with minimal FDG uptake, as this is a common site for reactive lymphadenopathy. On the other hand, there are additional small volume bilateral submental and submandibular lymph nodes (up to 6 mm short axis) with no definite FDG uptake. Uptake of the tracer elsewhere is within physiological limits.

We report a 19-year-old female, a known case of acinic cell carcinoma of the right parotid gland status after right parotidectomy. She first presented in 2017 with a complain of parotid swelling for one year. She did not have any family history of cancer or history of radiation exposure. The CT scan showed a right parotid gland lesion (Figure 1). Soon after that, a diagnostic parotidectomy was done in a local hospital on October 29, 2017. The histopathology revealed a multifocal acinic cell carcinoma of the right parotid gland T1, N1, M0 involving both deep and superficial lobes, with a margin of 1 mm, and 1 intraparotid lymph node was involved (Figure 2). Then, she was admitted electively to our tertiary care hospital. A PET-CT was done on 20 November, 2017, and showed a mild background level FDG avidity (SUVmax 2.2) in the region of the right parotid. This uptake is most likely postsurgical (Figure 3). Afterwards, a right total parotidectomy with unilateral supraomohyoid dissection was performed in December 2017. At that time, she started her radiotherapy, and the total dose that was prescribed for her is 6000 cGy. It was over 30 sessions covering the parotid area and the neck up to the midline. The patient completed her course in March 2018. Since then, she has been following regularly in our tertiary hospital.

**Figure 4:**
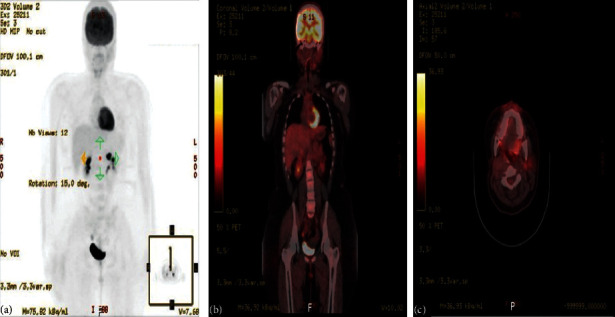
The PET scan in November 2018. The study was compared with the previous scan of November 20, 2017. Overall, the study ((a)–(c)) is stable without any evidence of local recurrence or distant metastasis.

**Figure 5:**
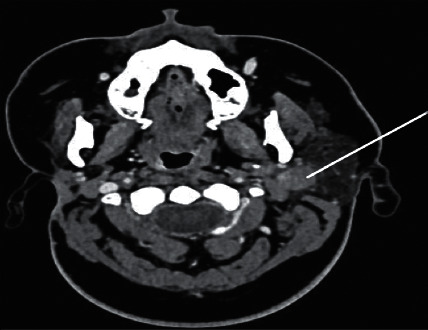
The CT scan showing a left deep parotid gland lesion in 2020.

**Figure 6:**
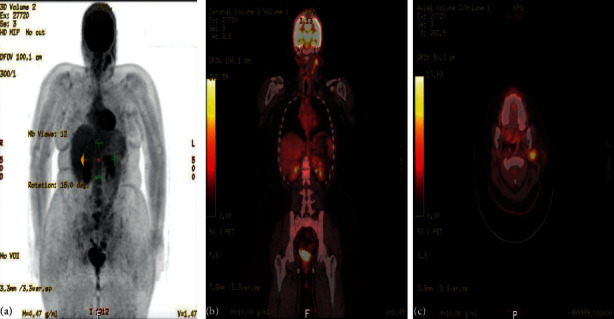
The PET scan in August 2020. The study showed an interval anatomic increase in size and metabolic progression in the known suspicious left deep parotid lesion ((a)–(c)). Measure approximately 2.0 × 1.9 × 2.2 cm in anteroposterior, transverse, and craniocaudal dimensions, respectively, with standard uptake value max (SUVmax) of 3.2. The SUVmax was previously 2.2. On the other hand, there is a more prominent left infraparotid subcentimeter node showing mild fluorodeoxyglucose (FDG) uptake. SUVmax 1.2 is indeterminate. Moreover, an anatomically stable mild FDG-avid left jugulodigastric subcentimeter node with SUVmax up to 1.4 and right supraclavicular node (SUVmax 1.0) are nonspecific, likely reactive. No other size significant FDG-avid cervical lymphadenopathy.

**Figure 7:**
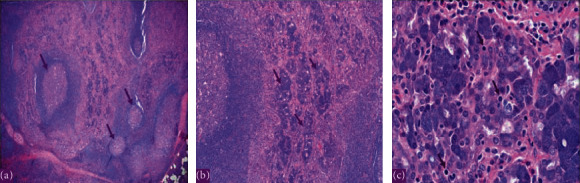
The histopathology with hematoxylin and eosin (H&E) stain showing acinic cell carcinoma with lymphoid background of the left parotid gland in 2020. (a) Low power showing lymphoid-rich stroma. (b) Medium power showing tumor with acini and granular cytoplasm. (c) High power x60 showing the basophilic granular cytoplasm (zymogen granules of neoplastic serous acini). The same tumor occurred metachronously in the left parotid gland.

**Figure 8:**
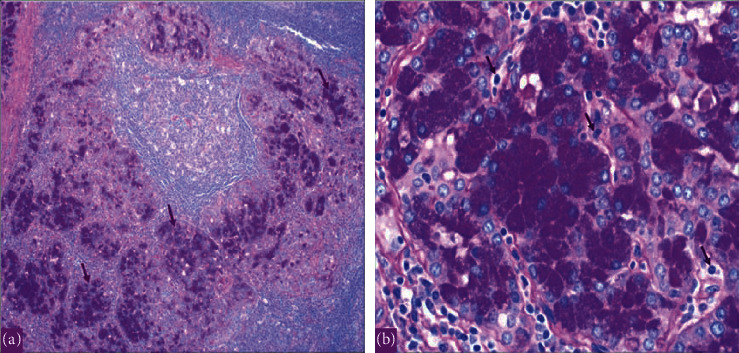
The histopathology with periodic acid–Schiff plus diastase (PASD) stain showing acinic cell carcinoma of the left parotid gland in 2020. (a) Periodic acid–Schiff plus diastase (PASD) stain low power showing acinic cell carcinoma cells with PASD-positive granular cytoplasm. (b) Periodic acid–Schiff plus diastase (PASD) stain high power showing PAS + diastase resistant granules in Acinic cells.

**Figure 9:**
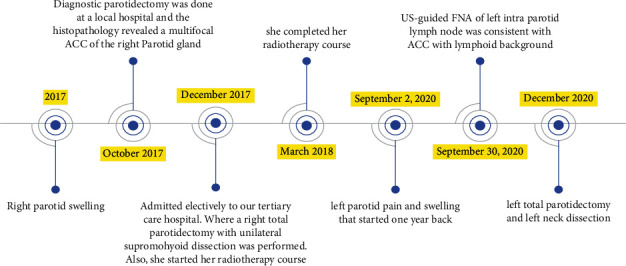
Symptoms, investigations, radiotherapy course, and surgeries.

**Figure 10:**
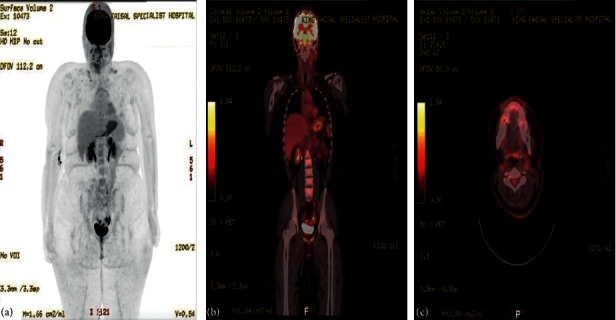
The PET scan in January 2022. The study ((a)–(c)) was compared with previous studies. The upper and lower cervical lymph nodes are showing mild FDG avidity with SUVmax of 1.8. However, there is no change morphologically, and it is most likely reactive. On the other hand, physiological radiotracer distribution is seen in the brain and there are no new suspicious FDG-avid lesions seen otherwise.

On 07 November, 2018, a PET-CT of the whole body was done and was compared with previous scans of the patient. Overall, the study is stable without any evidence of local recurrence or distant metastasis (Figure 4). Furthermore, on the 2^nd^ of September, 2020, the patient presented with left parotid pain and swelling that started one year back. On examination, a tender left parotid mass almost 2 cm in size was palpable. There were no skin changes over or surrounding the left parotid gland, and the facial nerve was intact bilaterally. The CT scan showed left deep parotid gland lesion (Figure 5). PET scan showed an anatomic increase in size and metabolic progression in the known suspicious left deep parotid lesion. Measure approximately 2.0 × 1.9 × 2.2 cm in anteroposterior, transverse, and craniocaudal dimensions, respectively, with standard uptake value max (SUVmax) of 3.2 (Figure 6). Moreover, the US-guided FNA of the left intraparotid lymph node was consistent with ACC with lymphoid background (Figure 7 and Figure 8) T1, N0, M0. The patient then refused any radiation on this side. A left total parotidectomy and left neck dissection (Figure 9) was done in December, 2020. Since then, the patient has been following regularly in our hospital for any signs of recurrence, and the latest PET-CT of the patient was done in January, 2022 (Figure 10).

## 3. Discussion

ACC is usually confined (92%) to the parotid gland [5]. It can recur after a prolonged period of time as in Grange's patient who developed recurrence after being free for 27 years [6]. ACC is the second most frequent form of parotid tumor in children and the most frequent form of bilateral malignant parotid tumors [7]. 3 out of 63 acinic cell carcinoma cases in Eneroth's series turned to be bilateral with an incidence of 4.8% [3]. Rarely, ACC can affect both parotid glands in a single individual. A bilateral ACC of the parotid gland could either coexist simultaneously at the same time, which is known as “synchronous tumor.” Or it could be developed consecutively over a certain period of time, which is known as “metachronous tumor.” Importantly, all ACC patients should have regular follow-up for both parotid glands to watch out for recurrence or a metachronous tumor [3].

**Figure 11:**
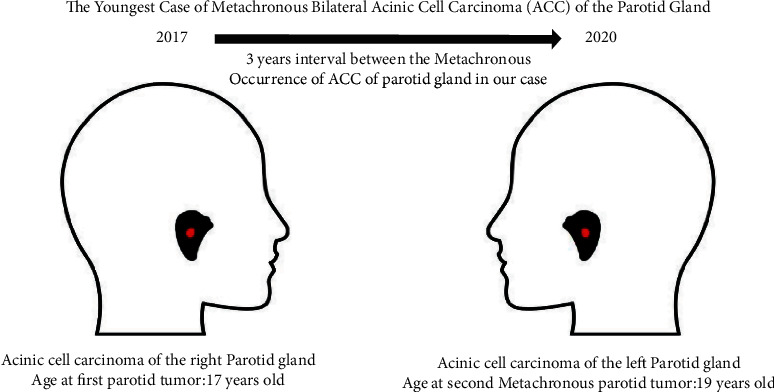
Representation of the bilateral metachronous occurrence of the parotid gland ACC in our case.

Our patient is a known case of ACC of the right parotid gland, which was resected in December, 2017. Then, she completed a radiotherapy course of 6000 cGy in March, 2018, and was cancer free for three years. In September, 2020, she presented with a complain of pain and swelling of the left parotid gland, which turned out to be an ACC of the left parotid gland. Furthermore, the following reasons support diagnosing our patient as a metachronous bilateral ACC of the parotid gland, instead of metastatic ACC from the right parotid gland to the left parotid gland. First, there is a three-year time gap between the initial right parotid tumor and the subsequent left parotid tumor, during which the patient completed her radiotherapy course and was cancer free, which indicates that these tumors are from a different time frame rather than metastatic from one another. Second, the lymphatic drainage of both parotid glands does not cross each other, thus making the idea of parotid gland tumor metastasizing to the other parotid gland is highly unlikely. Until now, only a few cases of bilateral tumor have been reported in the literature [2, 5–18], especially in regards to the metachronous bilateral ACC of the parotid gland, where the contralateral parotid gland developed a second primary cancer after a period of time. We have only found 5 cases of metachronous bilateral ACC reported in the literature that are summarized in Table 1. To our knowledge, our patient represents the sixth case of metachronous bilateral ACC of the parotid gland published in the English literature. Also, we report the youngest patient to develop metachronous bilateral ACC of the parotid gland (Figure 11).

## 4. Conclusion

In conclusion, despite the rareness of metachronous occurrence of bilateral ACC of the parotid gland, it is still encountered in the medical practice. This necessitates the importance of regular and careful follow-up for these patients. Periodic radiological investigations for both parotid glands are necessary to identify intraparotid small lesions before they acquire aggressive malignant potential.

## Figures and Tables

**Table 1:** All cases present in the literature reporting bilateral metachronous ACC of the parotid gland.

Sex	Age at presentation (first parotid tumor/second)	Metachronous occurrence	Reference
Female	17/19 (the youngest)	3 years interval	Alhayaza et al. (this case)
Female	26/37	11 years interval	Bartos [2]
Male	27/42	15 years interval	Eneroth et al. [3]
Female	35/36	1 year interval	Palma et al. [16]
Female	55/58	3 years interval	Nuutinen et al. [17]
Male	60/68	8 years interval	Turnbull et al. [18]

## Data Availability

The data used to support this study are included within the article and supplementary materials and are available from the corresponding author upon request.

## Abbreviations

ACC: Acinic cell carcinoma
FNA: Fine needle aspiration
PET: Positron emission tomography
CT: Computed tomography
US: Ultrasound.
